# Molecular Typing of *Mycoplasma pneumoniae* Strains in Sweden from 1996 to 2017 and the Emergence of a New P1 Cytadhesin Gene, Variant 2e

**DOI:** 10.1128/JCM.00049-19

**Published:** 2019-05-24

**Authors:** Karolina Gullsby, Björn Olsen, Kåre Bondeson

**Affiliations:** a Centre for Research and Development Uppsala University/Region Gävleborg, Gävle, Sweden; b Department of Medical Sciences, Section of Clinical Microbiology and Infectious Diseases, Uppsala University, Uppsala, Sweden; c Department of Medical Sciences, Zoonosis Science Centre and Infectious Diseases, Uppsala University, Uppsala, Sweden; University of Iowa College of Medicine

**Keywords:** MLVA, Mycoplasma pneumoniae, P1 typing, molecular typing

## Abstract

Mycoplasma pneumoniae causes respiratory infections, such as community-acquired pneumonia (CAP), with epidemics recurring every 3 to 7 years. In 2010 and 2011, many countries experienced an extraordinary epidemic peak. The cause of these recurring epidemics is not understood, but decreasing herd immunity and shifts in the strains’ antigenic properties have been suggested as contributing factors. M. pneumoniae PCR-positive samples were collected between 1996 and 2017 from four neighboring counties inhabited by 12% of Sweden’s population. A total of 578 isolates were characterized directly from 624 clinical samples using P1 typing by sequencing and multilocus variable number tandem repeat analysis (MLVA). A fluorescence resonance energy transfer (FRET)-PCR approach was also used to detect mutations associated with macrolide resistance in the 23S rRNA gene. Through P1 typing, the strains were classified into type 1 and type 2, as well as variants 2a, 2b, 2c, and a new variant found in nine of the strains, denoted variant 2e. Twelve MLVA types were distinguished, and 3-5-6-2 (42.4%), 4-5-7-2 (37.4%), and 3-6-6-2 (14.9%) predominated. Several P1 and MLVA types cocirculated each year, but type 2/variant 2 strains and MLVA types 3-5-6-2 and 4-5-7-2 predominated during the epidemic period comprising the peak of 2010 and 2011. In 2016 and 2017, type 1 became more common, and MLVA type 4-5-7-2 predominated. We also found that 0.2% (1/578) of the strains carried a macrolide resistance-associated mutation, indicating a very low prevalence of macrolide resistance in this region of Sweden.

## Abstract (precis)

Mycoplasma pneumoniae causes respiratory infections, such as community-acquired pneumonia (CAP), with epidemics recurring every 3 to 7 years. In 2010 and 2011, many countries experienced an extraordinary epidemic peak.

## INTRODUCTION

Mycoplasma pneumoniae is among the smallest free-living bacteria. It has a genome size of 0.816 Mbp and is regarded a genetically stable microorganism (1–3). Humans are the only host, and it causes primarily respiratory infections that range from mild upper respiratory infections to serious lower respiratory infections. Furthermore, M. pneumoniae may cause extrapulmonary manifestations, such as encephalitis, Stevens-Johnson syndrome, pericarditis, and hemolytic anemia (4).

M. pneumoniae does not have an ordinary cell wall; therefore, it is naturally resistant to beta-lactam antibiotics. Macrolides, tetracyclines, and fluoroquinolones are effective treatments for M. pneumoniae infections. Macrolide-resistant M. pneumoniae was first reported in Japan in 2000 and has been found in several other countries since then (5). In Europe, the resistance level is low at 0 to 10%, but up to 80 to 90% of strains are resistant in China and Japan (6–13). The mechanism of resistance is coupled with point mutations in the peptidyl transferase loop of domain V in the 23S rRNA gene (5, 14).

**FIG 1:**
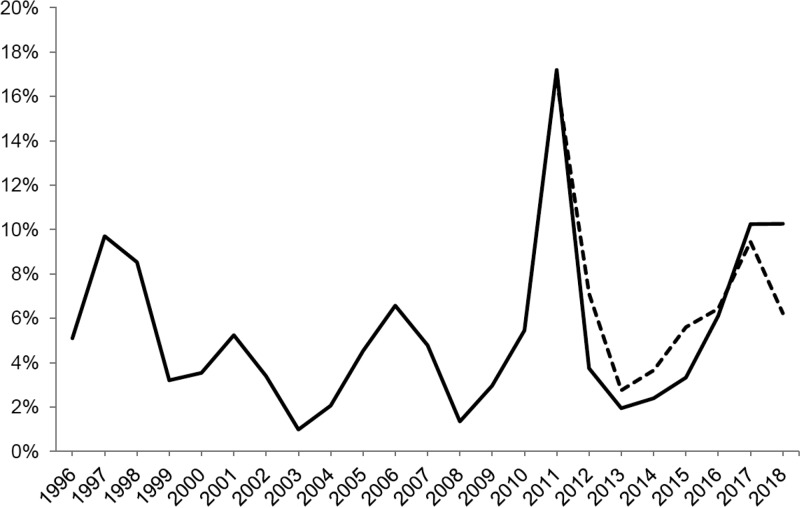
Proportion of PCR-positive M. pneumoniae samples per year in Sweden from 1996 to 2018. The black line is based on results from Gävle County (corresponding to approximately 1,000 to 1,500 samples analyzed per year). The dotted line is based on results from 13 counties in Sweden from 2011 to 2016 (corresponding to approximately 21,000 to 32,000 analyzed samples per year) and four counties in Sweden from 2017 and 2018 (corresponding to approximately 7,000 analyzed samples per year).

Every 3 to 7 years, M. pneumoniae epidemics increase, and in 2010 and 2011 there was an extraordinary epidemic peak in several countries, including Sweden (Fig. 1) (15–20). M. pneumoniae is not a notifiable disease in Sweden, so minimal information regarding regional and local outbreaks is known. However, laboratory reports indicate that there was another epidemic peak in 2017 (Fig. 1). The cause of these fluctuations is not fully understood, but recurring epidemic periods suggest antigenic shifts in strains, decreased herd immunity of the population, or both of these factors (21, 22). Molecular typing and identifying virulence factors of M. pneumoniae are important to further understand the epidemiology.

Traditionally, M. pneumoniae typing has been based on sequence differences in the P1 gene, which codes for a cytadhesin protein that acts as an important immunogen of the bacteria (4). The strains can be divided into two distinct types (i.e., type 1 and type 2) and further categorized into variants (variant 1 and variants 2a, 2b, 2c, and 2d) (23–29). Eight and ten copy variants of the repetitive sequences exist for repMP4 and repMP2/3, respectively. These are denoted as repMP4 a to h and repMP2/3 a to j, respectively, and are dispersed throughout the genome, where one of the copy variants is an integral part of the P1 gene (1, 30). Variants of the P1 gene are thought to evolve from intragenomic recombination events between repetitive sequences within the P1 gene and copy versions outside the gene (30).

A multilocus variable number tandem repeat analysis (MLVA) typing method was developed by Degrange et al. (31). The original MLVA method included repetitive sequences at five loci in the genome, but one of the loci, Mpn1, was later excluded due to an instability problem (18, 32). P1 and MLVA typing methods are often used complementarily and can be performed directly on clinical specimens. Other methods used to type M. pneumoniae include multilocus sequence typing, SNaPShot minisequencing, and whole-genome sequencing (WGS) (2, 3, 33, 34).

The aim of this study is to genetically characterize strains of M. pneumoniae that were collected over 21 years. P1 and MLVA typing methods, as well as a fluorescence resonance energy transfer (FRET)-PCR-based method for detecting mutations causing macrolide resistance, were applied using 624 patient samples collected in 1996 to 2017 from four counties in Sweden.

## MATERIALS AND METHODS

### Patient samples and control strains.

A total of 624 respiratory samples collected at four county hospitals in Sweden from 1996 to 2017 are included in this study. The samples came from patients where respiratory infection caused by Chlamydia pneumoniae or M. pneumoniae was suspected. Information related to the health care setting was known, but no further information regarding the clinical background of the patient was provided. Only one sample from each patient was included and duplicate samples (*n* = 10) were excluded. A total of 423 (67.8%) samples were collected from 2010 to 2013, which includes the epidemic peak in 2011. All of the samples were previously identified as positive for M. pneumoniae with real-time PCR at the respective clinical microbiological laboratories in Falun, Gävle, Karlstad, and Uppsala, Sweden. A total of 439 (70.4%) samples were collected in Gävle, and 185 (29.6%) were collected from the other three counties in 2012 to 2013. More than 95% were oropharyngeal and nasopharyngeal swab samples, and fewer than 5% were lower respiratory samples. Information regarding the patient’s age and sex, the sampling year, the county, and whether the sample was collected in an outpatient or inpatient setting was recorded for each sample before anonymization. The study was approved by the Regional Ethical Board in Uppsala, Sweden (Dnr 2014/292).

M. pneumoniae reference stains ATCC 29342 (Strain M129) and ATCC 15492 (Strain Mac) were used as positive controls. Four characterized macrolide-resistant strains harboring the A2063C, A2063G, A2064G, and C2617G mutations provided by Cécile Bébéar at the University of Bordeaux in France were used as positive controls during the macrolide resistance screening. Sterile water was used as a negative control.

The samples and control strains were extracted and treated as previously described using a MagNA Pure 96 (Roche Diagnostics, Basel, Switzerland) DNA and viral NA small volume kit, the Pathogen Universal program, and a starting volume of 200 μl (7).

### P1 typing.

P1 typing was performed by amplifying part of the repetitive sequence repMP2/3 within the P1 gene with a nested PCR and then sequencing as previously described by Dumke et al., with slight modifications (26). The first PCR, with a total volume of 50 μl, contained 10 μl of the DNA template, 0.5 μM concentrations (each) of the Mp5f and Mp16r primers (Eurogentec, Liège, Belgium), 200 nM concentrations of each deoxynucleoside triphosphate (dNTP), 1× LongRange PCR buffer (Qiagen, Hilden, Germany), and 2 U of LongRange PCR enzyme (Qiagen). Amplification was performed using the Rotorgene Q (Qiagen) and began with a heating step for 3 min at 93°C, followed by 40 cycles of 15 s at 93°C, 30 s at 58°C, and 3 min at 68°C, followed by an extension step of 10 min at 72°C. In the nested PCR, 10 μl of the PCR product from the first PCR was used as a template. The same composition and concentrations of the PCR mix was used, except that Mp11f and Mp14r were used as primers. The reaction mix was heated for 3 min at 93°C, and the cycling program included 40 cycles of 15 s at 93°C, 30 s at 58°C, and 1 min at 68°C, with a final step of 10 min at 72°C. The PCR product was sequenced using a BigDye Terminator v3.1 cycle sequencing kit (Thermo Fisher, Waltham, MA) and an ABI3730XL DNA Analyzer (Applied Biosystems, Foster City, CA). In order to discriminate between variants 2a and 2c, the strains where further analyzed with a nested PCR that targeted part of the repetitive sequence, repMP4, within the P1 gene (27). The composition of the PCR and cycling conditions were the same as those used to type the repMP2/3 sequence, except for the primers (unpublished sequences, kindly provided by Roger Dumke, Institute of Medical Microbiology and Hygiene, Dresden, Germany).

### Multiple-locus variable-number tandem-repeat analysis.

The method used for MLVA typing, which included five loci (i.e., Mpn1, Mpn13, Mpn14, Mpn15, and Mpn16), was performed as described previously by Degrange et al., with slight modifications (31). The amplifications were performed in two mixes: mix 1 contained 1× PCR buffer (Qiagen), 200 nM concentrations of each dNTP, 1 mM MgCl_2_; 0.5 μM concentrations (each) of primers Mpn1-F and Mpn1-R, 0.6 μM concentrations (each) of Mpn14-F, Mpn14-R, Mpn16-F, and Mp16-R, and 2.5 U of HotStarTaq enzyme (Qiagen). Mix 2 contained 1× PCR buffer, 200 nM concentrations of each dNTP, 1.5 mM MgCl_2_, 0.6 μM concentrations of each primer Mpn13-F, Mpn13-R, Mpn15-F, and Mpn15-R, and 2.5 U of HotStarTaq enzyme. The primers were obtained from Eurogentec (Liège, Belgium). The cycling conditions were as described with the exception that the number of cycles was extended to 40 (31).

The PCR products were mixed with GeneScan 500 Rox size standard (Applied Biosystems) and Hi-Di formamide (Applied Biosystems). Subsequent fragment size separation and a determination of the number of repeats at each locus were performed via capillary electrophoresis using an ABI3730XL DNA analyzer (Applied Biosystems). The fragment analysis was performed using PeakScanner 2 (Applied Biosystems) software, following the guidelines proposed by Chalker et al. (32).

### Macrolide resistance.

Macrolide resistance screening of the samples from 1996 to 2013 had been performed within a previous study (7). Screening of the additional 61 samples from 2016 and 2017 was performed using the same duplex FRET real-time PCR method targeting the 23S rRNA gene developed by Peuchant et al. (35). To verify the results, Sanger sequencing was performed on the PCR product from the control strains and a portion of the samples using a BigDye Terminator v3.1 cycle sequencing kit (Thermo Fisher) and an ABI3730XL DNA analyzer (Applied Biosystems).

### Data analyses.

The P1 gene sequences were aligned with the reference sequences that corresponded to the different types and variants of M. pneumoniae using ClustalW version v.1.4 in BioEdit v.7.2.5. The following reference strains and GenBank accession numbers were used during the alignment: strain M129 (type 1, M18639), strain Mac (type 2, AF290001.1), variant 1 (AF290000.1), variant 2a (AB024618.1), variant 2b (DQ383277.1), variant 2c (JN048895), and variant 2d (EF656612).

To visualize the MLVA typing results, the repeat numbers were recorded, and a minimum spanning tree analysis was performed using the default settings in Bionumerics v.7.6.2 (bioMérieux, Marcy l’Etoile, France). The results of the Mpn1 loci were omitted when analyzing the results, as proposed by the established guidelines (32).

The discriminatory powers for each typing method, P1 typing and MLVA were calculated using the Hunter-Gaston diversity index (HGDI) (36).

The strain frequencies of P1 and the MLVA types were compared over the epidemic periods. An epidemic period was defined as the year before the peak year until the beginning of the next peak (Fig. 1). Since very few strains were available during the first two epidemic periods, these were combined into one period (1996 to 2004). A Poisson probability test was calculated to determine whether a type or variant predominated significantly during an epidemic period, using the web-based program StatTools.

## RESULTS

### Patient samples.

The median age of the patients was 31 years (range, 1 to 91 years): 331 (53.0%) were women, and 293 (47.0%) were men. A total of 432 (69.2%) patients were from an outpatient setting, and 172 (27.6%) were collected in an inpatient setting. The clinical setting was unknown for 20 (3.2%) samples.

### P1 typing and MLVA.

**FIG 2:**
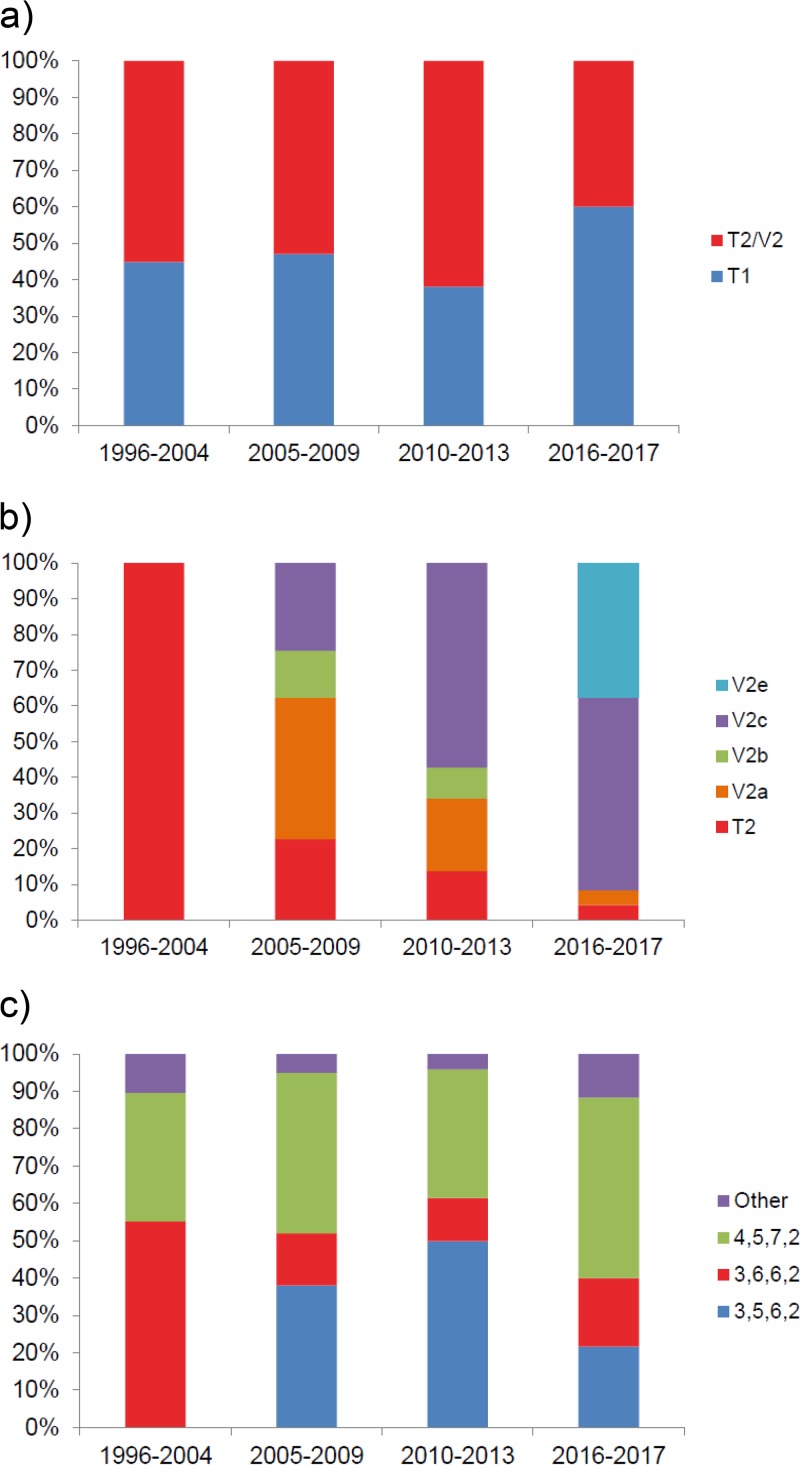
Distributions of P1 and MLVA types during different epidemic periods. (a) Distribution between type 1 and type 2/variant 2 strains; (b) distribution between type 2 and different variant 2 strains; (c) distribution of MLVA types.

**FIG 3:**
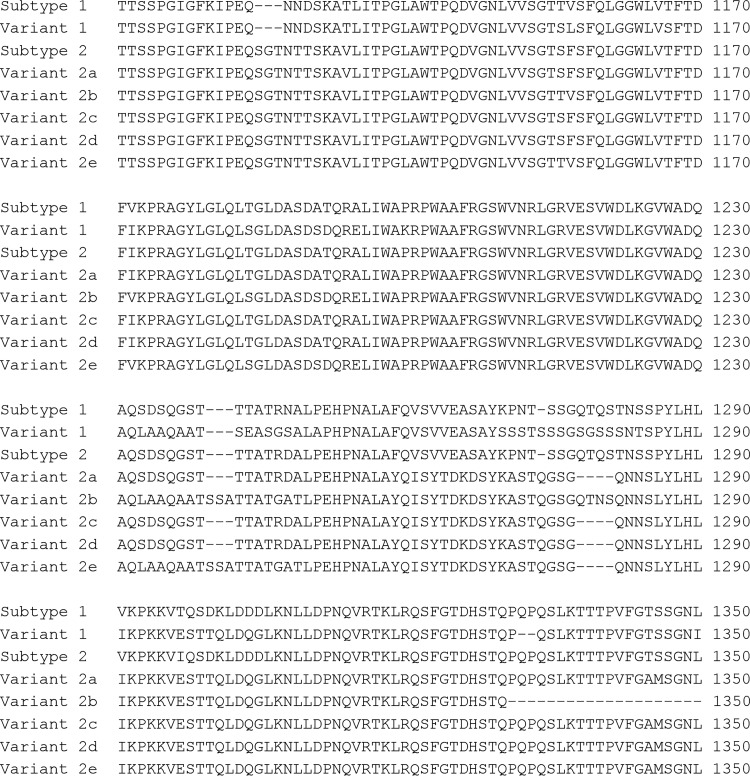
Differences of the amino acid sequences, which are part of the repMP2/3 within the P1 protein, between type 1 (M18639), variant 1 (AF290000.1), type 2 (AF290001.1), variant 2a (AB024618.1), variant 2b (DQ383277.1), variant 2c (JN048895), variant 2d (EF656612), and the newly discovered variant 2e (MK330954). The amino acid positions correspond to the P1 gene of M. pneumoniae, M129 (NC_000912.1).

Of the 624 samples, 578 (92.6%) were successfully analyzed using both typing methods. Both P1 types cocirculated during all of the epidemic periods, but there was a higher prevalence of type 2/variant 2 strains during the epidemic period of 2010 to 2013 (62.0%, *P* = 0.0007) (Table 1 and Fig. 2a). After 2005, variants of type 2 strains were more common than type 2 strains, and variant 2a predominated during the epidemic period of 2005 to 2009 (39.6%, *P* = 0.0297), and variant 2c predominated during the epidemic periods of 2010 to 2013 (57.3%, *P* < 0.0001) and 2016 and 2017 (54.2%, *P* = 0.0014) (Table 1 and Fig. 2b). In 2016 and 2017, nine strains showed a new deletion of 12 nucleotides within a sequence that was homologous to variant 2b, which is the same deletion carried by the variant 2a strains at that position, leading to a loss of four amino acids (Fig. 3). Sequencing of the full repMP2/3 element within the P1 gene showed that the new variant, designated variant 2e, was homologous to variant 2b (DQ383277.1) except for the 12-nucleotide deletion. As seen in variants 2a and 2b, all nine variant 2e strains also displayed a repMP4 element that was homologous to type 2 (data not shown). No variant 1 strains were found, and because of that the variable regions that were distinctive for variant 2d were not covered in this typing method; these strains could not be detected among the 71 variant 2a strains.

**TABLE 1:** Molecular typing results of P1 typing and MLVA for 578 M. pneumoniae samples isolated between 1996 and 2017 in Sweden

Yr	No. of samples	P1 type or variant^*a*^						MLVA			
		T1	T2	V2a	V2b	V2c	V2e^*b*^	3,5,6,2	3,6,6,2	4,5,7,2	Other
1996	2	2	0	0	0	0	0	0	0	2	0
1998	9	3	6	0	0	0	0	0	6	3	0
2001	9	3	6	0	0	0	0	0	6	2	1
2002	8	5	3	0	0	0	0	0	3	3	2
2004	1	0	1	0	0	0	0	0	1	0	0
2005	20	3	7	3	1	6	0	13	4	2	1
2006	35	21	3	3	4	4	0	7	7	20	1
2007	26	13	2	6	2	3	0	9	3	11	3
2008	8	5	0	3	0	0	0	3	0	5	0
2009	11	5	0	6	0	0	0	6	0	5	0
2010	29	7	5	7	0	10	0	17	5	4	3
2011	135	35	15	27	6	52	0	84	15	35	1
2012	178	89	9	15	10	55	0	73	16	82	7
2013	47	17	4	0	5	21	0	20	9	13	5
2016	28	15	1	0	0	11	1	11	2	14	1
2017	32	21	0	1	0	2	8	2	9	15	6
Total	578	244	62	71	28	164	9	245	86	216	31

a T, type; V, variant.

b V2e is a new variant detected in this study.

**FIG 4:**
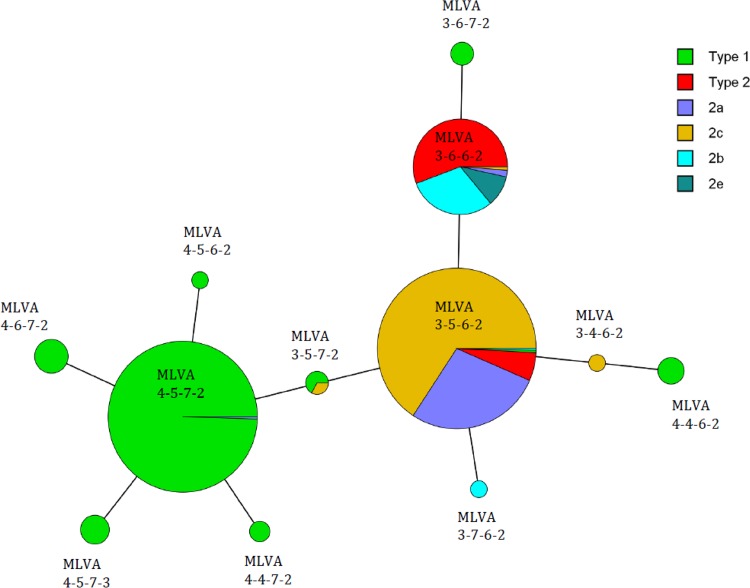
Minimum spanning tree of 578 M. pneumoniae isolates from four counties in Sweden from 1996 to 2017. Each circle denotes an MLVA type. The size of the circle is proportional to the number of isolates belonging to each MLVA type, and the color corresponds to the P1 type. The distance between MLVA types corresponds to the number of allelic changes, and each line represents one allelic change.

Thirty-eight MLVA types were identified using five loci, including Mpn1 (data not shown). When the Mpn1 loci were excluded according to the new guidelines, 12 MLVA types remained, including, to our knowledge, two new types: 3-7-6-2 and 4-4-6-2 (Fig. 4). The MLVA results for the new types were confirmed through repeated testing, but unfortunately, not enough material was available for further verification via sequencing. The most common MLVA types were 3-5-6-2 (*n* = 245), 4-5-7-2 (*n* = 216), and 3–6-6-2 (*n* = 86), which comprised 94.6% of the strains (Table 1). Only 31 (5.4%) strains belonged to the other nine MLVA types, with only one or two strains at most of each type per year. During the epidemic period of 1996 to 2004 MLVA type 3-6-6-2 predominated (55.2%, *P* = 0.0381) (Fig. 2c). MLVA type 3-5-6-2 was first detected in 2005, which coincided with the introduction of the variant 2 strains. During the epidemic periods from 2005 to 2009 and 2010 to 2013, both MLVA types 3-5-6-2 and 4-5-7-2 predominated (38.0% [*P* = 0.0092] and 43.0% [*P* = 0.0007], respectively, 49.9% [*P* < 0.0001] and 34.4% [*P* = 0.0002]) (Fig. 2c). During the last epidemic period of 2016 to 2017, MLVA type 4-5-7-2 predominated (48.3%, *P* = 0.0009) (Fig. 2c). With a few exceptions, the MLVA types correlated to either P1 types 1 or 2 (Fig. 4). One variant 2a strain was of MLVA type 4-5-7-2, while one type 1 strain was of MLVA type 3-5-6-2. Moreover, two type 1 strains and one variant 2c strain had MLVA type 3-5-7-2. All of the new variant 2e stains were of MLVA type 3-6-6-2, as were most of the variant 2b strains.

The HGDI values calculated for these samples were 0.71 for the P1 typing and 0.66 for the MLVA method.

### Macrolide resistance.

In the previous study, no macrolide-resistant M. pneumoniae were detected in the 563 samples collected from 1996 to 2013 (7). When analyzing the additional 61 samples from 2016 and 2017, one sample showed a melting temperature (*T_m_*) that was concordant with the control strains that harbored either the A2063C or A2063G mutation (*T_m_*, 49°C). Sequencing verified that the strain had an A2063G mutation, which has been shown to provide a high level of macrolide resistance (14). The strain was determined to belong to P1 type 1 with the MLVA 4-5-7-3 profile and came from a 16-year-old male patient visiting an outpatient facility. A correlation of recorded melting temperature profiles of the control strains and patient-derived strains to target sequences showed sequences as expected.

### Data availability.

The sequences of the novel repMP2/3 elements reported in this study are listed in the GenBank under accession numbers MK330954, MK330955, MK330956, MK330957, MK330958, MK330959, MK330960, MK330961, and MK330962.

## DISCUSSION

This study characterized 578 M. pneumoniae strains collected in Sweden from 1996 to 2017 by applying molecular methods for P1 typing, MLVA, and the detection of macrolide resistance.

A polyclonal distribution of strains was observed, even during the years in which M. pneumoniae epidemics occurred, which are in agreement with previous typing studies (9, 13, 20, 37, 38). However, in our region, there was a higher prevalence of type 2/variant 2 strains during the epidemic period, including the extraordinary epidemic outbreak of 2010 and 2011. These results are consistent with research conducted in Slovenia but contradict findings in France, England, Japan, and China, where type 1 strains dominated during these epidemic years (6, 9, 13, 19, 39). Studies from Germany and the United States show an almost equal distribution of both types during the epidemic peak (20, 38). Therefore, it is clear that the type distribution pattern is not consistent over larger geographical areas. A shift in the predominance of variant 2 strains could be seen where variant 2a predominated from 2005 to 2009, and variant 2c predominated from 2010 to 2013 and in 2016 and 2017. In 2017, the proportion of M. pneumoniae-positive samples increased to about 10% in Sweden (Fig. 1), which implies a new epidemic peak. Interestingly, in 2016 and 2017, the proportion of type 1 strains increased from 38.0% in the previous period to 60.0%, and we recorded the emergence of a new genotype, denoted as variant 2e. In 2017, variant 2e was the second most prevalent genotype, comprising 8/32 (25.0%) of all strains and 8/11 (72.7%) of type 2/variant 2 strains. These findings may indicate a shift from variant 2c, which was the predominant type 2/variant 2 strain in the previous epidemic period, to variant 2e.

**FIG 5:**
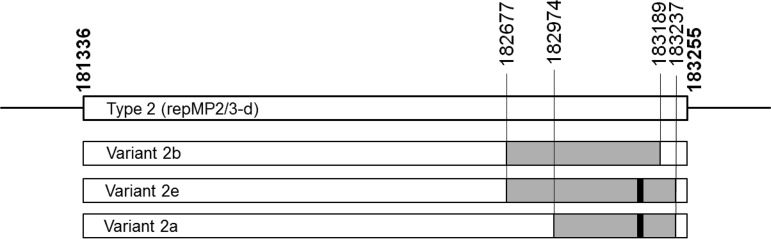
Schematic figure of the major differences between the repMP2/3 elements situated within the P1 adhesion gene. Variants 2a, 2b, and 2e are compared to type 2 according to the position of strain Mac (CP010550.1). The gray bars indicate the inserted parts of the repMP2/3-a element that characterize each of the variant strains. The insertions are identical to the corresponding positions: 128382 to 128906 (variant 2b, DQ383277.1), 128382 to 128954 (variant 2e, MK330954), and 128688 to 128954 (variant 2a, AB024618.1), except for 12 bp, which shows a deletion at positions 128751 to 128762 (indicated by a black mark) in variants 2a and 2e.

The repMP2/3 sequence was identical in all nine variant 2e strains and closely resembled the sequence of variant 2b, except for the deletion of 12 nucleotides, which led to the loss of four amino acids. The recurrence of this specific deletion within two P1 variants (i.e., variants 2a and 2e) may provide a selective advantage resulting in higher prevalence during epidemics. The sequence similarities between other repMP2/3 elements within the genome indicate that the new variant 2e is likely to be a product of rearrangement in which part of the repMP2/3-a element is inserted into the repMP2/3-d element, which is situated in the P1 gene (Fig. 5). The same mechanism behind the development of variants, such as 2a and 2b, has previously been suggested by Spuesens et al. (30).

Unfortunately, there is no information available regarding the severity of the infection or the treatment of patients infected with the strains described in this study. Interestingly, all of the patients infected with the new variant 2e were hospitalized, including two patients who were treated in an intensive care unit, which indicates that they were suffering from a more serious disease. Several studies have found no correlation between a particular genotype and disease severity (40, 41). However, other studies indicate that there could be differences in the virulence of the type 1 and type 2 strains. Rodman Berlot et al. showed that children infected with M. pneumoniae type 2 strains had a higher C-reactive protein level and were more often hospitalized, but extrapulmonary manifestations developed in equal proportions regardless of type (42). In proteome studies conducted by Lluch-Senar et al., type 2 strains expressed higher levels of the community-acquired respiratory distress syndrome toxin, which is one of the major virulence factors in M. pneumoniae (43). Simmons et al. showed that type 2 strains form a more robust biofilm that could give them increased resistance to antibiotic treatment and might allow them to evade the immune response (44).

A higher HGDI rate was calculated for the P1 typing method than for the four loci MLVA method. Removing the Mpn1 loci greatly reduced the discriminatory power of the method. Most of the strains belonged to MLVA types 3-5-6-2, 4-5-7-2, and 3-6-6-2, which seemed to be the most common types globally, except in China and Japan, where MLVA type 3-6-6-2 is rare but 4-5-7-3 is common (10, 11, 13, 19, 38, 45, 46). In order to improve the diversity index of the MLVA method, the inclusion of other loci should be considered. Recently, Zhang et al. have identified new loci with variable-number tandem repeats, which could be included in a future MLVA scheme to improve the discriminatory performance of the method (47).

In the previous study, 563 strains were screened for mutations associated with macrolide resistance, but no mutations were found (7). However, in the present study, one sample with the A2063G mutation was detected. Information on whether the patient had been treated previously with macrolides was not available. The sample was collected at an outpatient facility, and the patient had not been tested previously for M. pneumoniae; therefore, the sampling was assumed to have taken place before treatment was initiated. In Sweden, antibiotics, including macrolides, are used under strict indications, which is reflected in the lowest consumption of macrolides in Europe (48, 49). One case of macrolide-resistant M. pneumoniae after treatment with a macrolide has been described previously in Sweden (50). Thus, at this point, only sporadic cases of macrolide resistance in M. pneumoniae have been detected.

The lack of cultured strains makes the phenotypic detection of resistance impossible and complicates WGS typing. However, current WGS studies confirm that M. pneumoniae is a highly genetically stable species and the classification of strains into type 1 and type 2 is still valid, and they represent two separate lineages (2, 3, 43). Major variations in the genome can be seen in the P1 gene and ORF6, which also represents part of the adhesion complex, making the P1 gene a target for typing, although it has low discriminatory power, still useful. In our study, 92.6% of the samples could be analyzed using all three methods. Forty-six of the samples could not be fully analyzed, which was likely due to a small amount of DNA in the sample, repeated freeze-thaw cycles, and prolonged storage.

In conclusion, we detected the cocirculation of different M. pneumoniae types over a 21-year period and found that type 2/variant 2 strains predominated during the epidemic period from 2010 to 2013. The variant 2 strains replaced the type 2 strains after 2005, and there is a shift from variant 2a to variant 2c strains between 2005 to 2009 and 2010 to 2013. A new variant, denoted variant 2e, was detected in 2016 and 2017. Only one of the strains had a mutation associated with macrolide resistance, but surveillance must continue due to the global increase in antibiotic resistance. Aside from the postepidemic decrease in M. pneumoniae-specific immunity within the population, it is unclear whether the introduction of new variants with the potential to display an altered set of immunodominant epitopes could lead to a rise in epidemics.
